# Chronic pain is common in mitochondrial disease

**DOI:** 10.1016/j.nmd.2020.02.017

**Published:** 2020-05

**Authors:** Jelle van den Ameele, Joshua Fuge, Robert D.S. Pitceathly, Sarah Berry, Zoe McIntyre, Michael G. Hanna, Michael Lee, Patrick F. Chinnery

**Affiliations:** a Department of Clinical Neurosciences, University of Cambridge, Cambridge Biomedical Campus, Cambridge CB2 0QQ, UK; b WT/CRUK Gurdon Institute, Tennis Court Road, Cambridge CB2 1QN, UK; c Department of Neuromuscular Diseases, UCL Queen Square Institute of Neurology and The National Hospital for Neurology and Neurosurgery, London WC1N 3BG, UK; d Division of Anaesthesia, University of Cambridge, Cambridge CB2 2QQ, UK; e MRC Mitochondrial Biology Unit, University of Cambridge, Cambridge CB2 0XY, UK

**Keywords:** Mitochondrial disorders, Pain, Neuropathy, Mitochondria, Genetics

## Abstract

In the absence of cure, the main objectives in the management of patients with mitochondrial disease are symptom control and prevention of complications. While pain is a complicating symptom in many chronic diseases and is known to have a clear impact on quality of life, its prevalence and severity in people with mitochondrial disease is unknown. We conducted a survey of pain symptoms in patients with genetically confirmed mitochondrial disease from two UK mitochondrial disease specialist centres. The majority (66.7%) of patients had chronic pain which was primarily of neuropathic nature. Presence of pain did not significantly impact overall quality of life. The m.3243A>G *MTTL1* mutation was associated with higher pain severity and increased the likelihood of neuropathic pain compared to other causative nuclear and mitochondrial gene mutations. Although previously not considered a core symptom in people with mitochondrial disease, pain is a common clinical manifestation, frequently of neuropathic nature, and influenced by genotype. Therefore, pain-related symptoms should be carefully characterised and actively managed in this patient population.

## Highlights

• Chronic pain is common in patients with mitochondrial disease.
• Pain due to mitochondrial disease is primarily of neuropathic nature.
• Distribution, intensity and type of pain are genetically determined.

## 1 Introduction

Mitochondrial diseases are genetic disorders caused by mutations in the mitochondrial and nuclear genomes that cause dysfunction of oxidative phosphorylation and affect approximately 1 in 5000 people in the UK [1]. Curative treatments are currently lacking and management is largely based on symptomatic therapies and maximizing quality of life [2,3]. Pain has been reported in series of patients with mitochondrial disease, related to myopathy [4], neuropathy [5] and headache [6]. However, the prevalence, severity, impact on the quality of life and the genetic predisposition of chronic pain in this population is not fully known. Based on anecdotal experience of patients frequently reporting chronic pain symptoms, we conducted a service evaluation to survey pain in patients with genetically determined mitochondrial disease attending two specialist mitochondrial disease clinics in the UK.

## 2 Methods

We conducted a postal survey as part of a service evaluation of all adults with genetically confirmed mitochondrial disease who attended the clinic at two different centres in Cambridge and London during 2018 and the first half of 2019. The service evaluation had ethical approval by the Institutional Clinical Governance and Audit Board. The pain-related questionnaires included the first 6 questions of the Brief Pain Inventory, PainDETECT, SF12-v2, and Small Fibre Neuropathy screening list (SFNSL). Statistical analyses were performed in *R* and the SF12v2 ProCore analysis software. Normality and equality of variance were tested with Shapiro and Bartlett tests respectively. Significance tests are indicated in the text or figure legends. All data is included in the manuscript and figures. All data from the survey is available in Supplementary Table A.2.

## 3 Results

### 3.1 Prevalence and nature of chronic pain in adults with mitochondrial disease

**Fig. 1:**
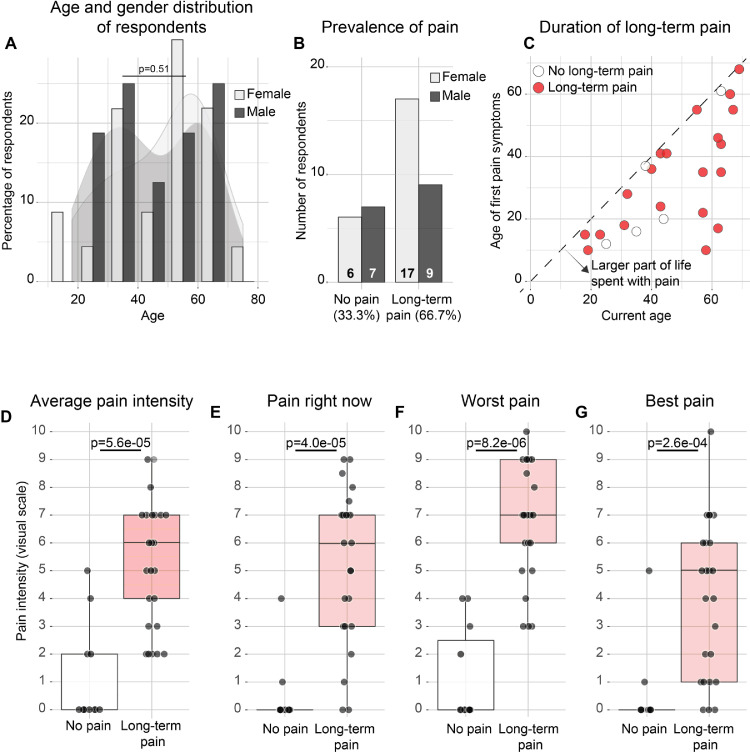
Patients with mitochondrial disease experience chronic pain. (A) Number of female (light grey) and male (dark grey) respondents according to age. *P* = 0.51 for the age distribution of female and male respondents (*t*-test). (B) Number of female and male respondents who reported chronic pain (i.e., long-term pain other than minor headaches, sprains and toothaches, in the last six months). (C) Age at which respondents first experienced chronic pain versus current age. Dashed line is where age of onset is same as current age. (D–G) Average, current, worst and best pain intensities are significantly higher among those respondents who reported chronic pain (Wilcoxon test).

**Fig. 2:**
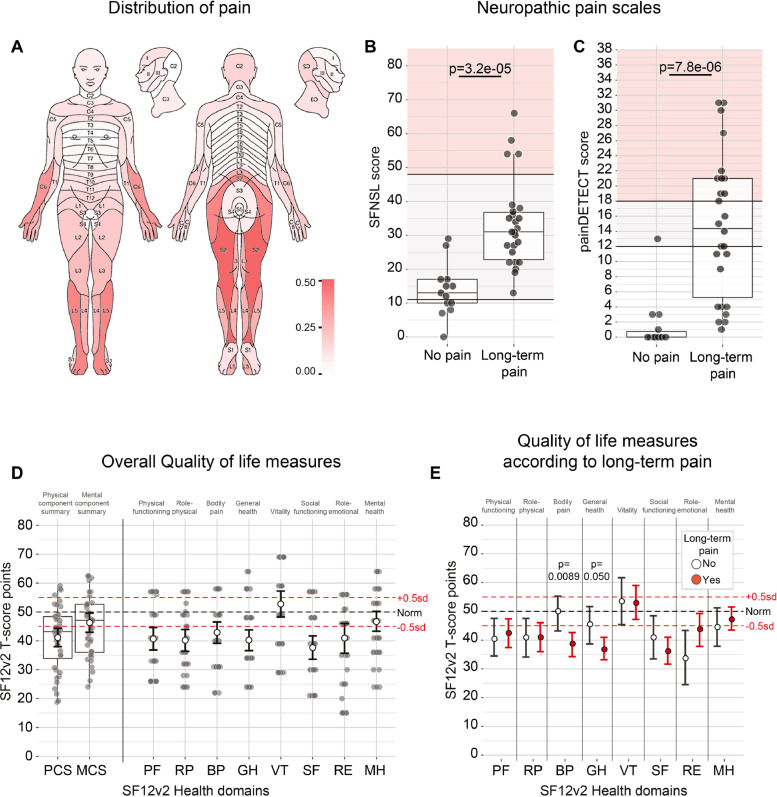
Chronic pain often has a neuropathic character. (A) Distribution of pain across the body. Dermatomes are shaded according to proportion of respondents who reported pain in this area. (B,C) SFNSL (B) and painDETECT (C) scores are significantly higher amongst those respondents who reported chronic pain (*t*-test, B; Wilcoxon test, C). Grey shaded area indicates possible neuropathic pain; red shaded area indicates likely neuropathic pain. Box-and-whisker plots depict median, interquartile range (box) and 1.5IQR below and above the first and third quartiles respectively (whiskers). Datapoints indicate individual responses. (D) SF12v2 quality of life compound (left) and subdomain (right) measures presented as t-scores compared to the general population, with 50 being the norm and every point equivalent to 0.1 standard deviation (sd) from the norm. White dots are the mean of the respondents, with the error bars representing the 95% confidence interval around the mean calculated through bootstrapping of non-parametric values. Datapoints indicate individual responses. (E) Only the bodily pain subdomain score is significantly different between respondents with (white) or without (red) chronic pain (Wilcoxon test).

Out of the 66 patients contacted, 39 patients (59.1%; 23 females and 16 males between ages 18 and 75) completed and returned the surveys. Patient demographics and genetic diagnoses of responders are listed in Table 1 and Fig. 1A; non-responders are listed in Table A.1. One questionnaire was excluded, because the first question about presence of chronic pain was not answered. All data are available in Table A.2. The majority (26/39, 66.7%) had experienced chronic pain in the last six months, distinct from minor headaches, sprains and toothaches (Fig. 1B). This is significantly higher than the general population in the UK (*p* = 0.00034; Fisher's exact test), where about 1/3 (3202/8599) reports chronic pain lasting longer than 3 months [7]. There was no significant difference between men and women affected by chronic pain (*p* = 0.49; Fisher's exact test; Fig. 1B), but respondents with chronic pain were on average older (51.5 ± 15.9 years with and 38.4 ± 13.1 years without chronic pain; *p* = 0.022; Wilcoxon test; figure A.1A). Age of onset ranged from 10 to 68 years (Fig. 1C), which meant that respondents on average had experienced pain for 31.3% of their lives (range 0%–82.8%; s.d. 23.8; Fig. A.1B). The average severity of pain among respondents with chronic pain was 5.4 ± 2.7 on a scale of 0–10 (Fig. 1D–G). On average 15.9 ± 21.1 dermatomes (out of 120: 30 on either side, front and back of the body) were affected by chronic pain (Fig. A.2A). Pain was predominantly located in the lower back, and in the peripheries of the upper and lower limbs (Fig. 2A). The pain in the distal limbs suggests a neuropathic component to the pain mechanism, consistent with the high overall scores on the painDETECT and SFNSL questionnaires (Fig. 2B,C; Fig. A.2B). All patients with chronic pain (26/26) had a positive or ambiguous score for the presence of small-fibre neuropathy on the SFNSL questionnaire (Fig. 2B), as did more than half of the patients (14/26) on the painDETECT questionnaire (Fig. 2C), together indicating an underlying neuropathy as the likely source of pain in many patients.

**Table 1:** Demographics and genetic diagnosis of patients (*N* = 39) included in the survey.

Age – average years (s.d.)	47.1 (16.1)
Gender – no. (%)	
Male	13 (33.3)
Female	26 (66.7)
Type of mutation – no. (%)	
Mitochondrial	30 (76.9)
Nuclear	9 (23.1)
Genetic mutation – no. (%)	
m.3243A>G	17 (43.6)
Single deletion	4 (10.3)
m.14487T>C	3 (7.7)
SPG7	3 (7.7)
POLG	2 (5.1)
m.13513G>A	1 (2.6)
m.14430A>G	1 (2.6)
m.16021-16022del	1 (2.6)
m.3260A>G	1 (2.6)
m.8344A>G	1 (2.6)
m.8993C>T	1 (2.6)
OPA1	1 (2.6)
RNASEH1	1 (2.6)
TWNK	1 (2.6)
TYMP	1 (2.6)

### 3.2 Effect of chronic pain on quality of life

Patients had overall lower scores on most quality of life measures of the SF12v2 compared to the general population (Fig. 2D). However, pain was not a major contributing factor since those who reported long-term pain did only score worse on the Bodily Pain (BP) and General Health (GH) subdomain of the SF12v2, but not on the other measures (Fig. 2E). Average pain intensity, painDETECT and SFNSL scores were most significantly correlated with the BP and GH measures, but not or inversely correlated with the other compound wellbeing scores (figure A.2C). When asked about their current medications, respondents who experienced chronic pain reported taking on average 0.88 ± 1.13 medications for pain relief. Of the 12/26 respondents with chronic pain who took pain medication, only 6 took one of the 4 NICE-recommended medications for neuropathic pain (gabapentin, pregabalin, duloxetine or amitriptyline) [8]. Altogether, these results suggest that despite the high prevalence of pain, the symptoms did not have a significant impact on the quality of life in these patients.

### 3.3 Genetic determinants of chronic pain

**Fig. 3:**
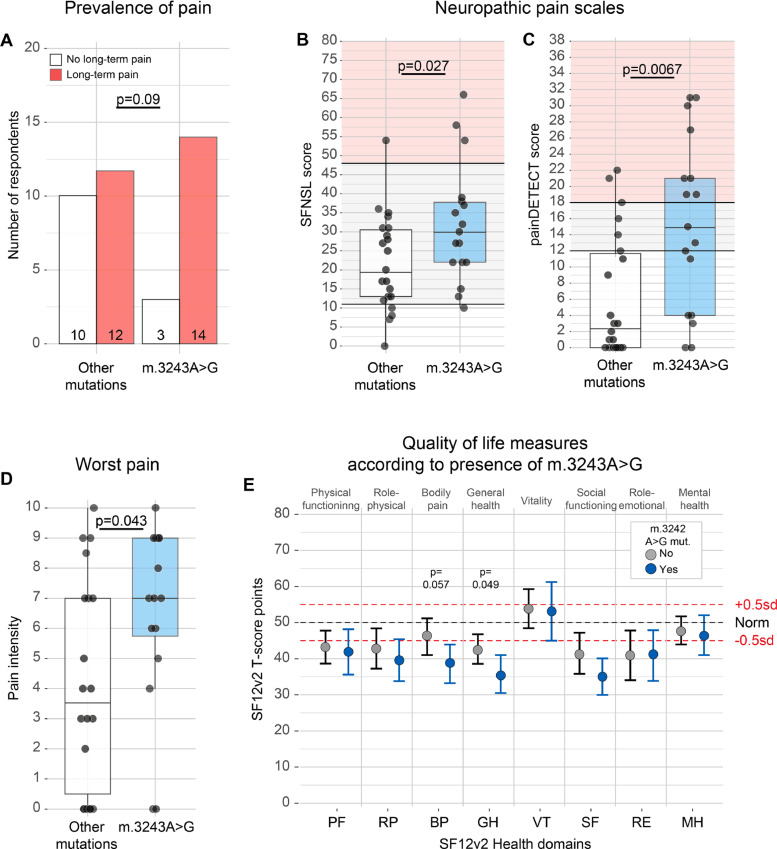
Chronic pain in mitochondrial disease is genetically determined. (A) Number of respondents with (red) or without (white) chronic pain who have an m.3243A>G mutation (Fisher's exact test). (B,C) SFNSL (B) and painDETECT (C) scores are significantly higher among those respondents who have an m.3243A>G mutation (*t*-test, B; Wilcoxon test, C). Grey shaded area indicates possible neuropathic pain; red shaded area indicates likely neuropathic pain. Box-and-whisker plots depict median, interquartile range (box) and 1.5IQR below and above the first and third quartiles respectively (whiskers). Datapoints indicate individual responses. (D) Worst pain intensity in patients with (blue) or without (white) the m.3243A>G mitochondrial mutation (Wilcoxon test). (E) SF12v2 subdomain scores in respondents with (blue) or without (grey) the m.3243A>G mutation (Wilcoxon test). 95% confidence interval around mean SF12v2 t-scores was calculated through bootstrapping of non-parametric values in *R*.

While all mitochondrial diseases primarily affect oxidative phosphorylation, different mutations can be associated with specific phenotypes [9]. Mitochondrial disease can be caused by mutations in the nuclear or in the mitochondrial genome. When comparing nuclear versus mitochondrial encoded mutations, we did not observe a difference in prevalence of chronic pain (*p* = 0.69; Fisher's exact test), average pain intensity (*p* = 0.52; Wilcoxon test), or scores on the neuropathy questionnaires (Fig. A.3A–C). Because of the large diversity of mutations in this patient group, we only had the power to test the role of the most common m.3243A>G *MTTL1* mutation (Table 1), which is typically associated with the MELAS (Mitochondrial Encephalopathy with Lactic Acidosis and Stroke-like episodes) or MIDD (maternally inherited deafness and diabetes) syndromes, but may also result in peripheral neuropathy [10]. The number of respondents who reported chronic pain was not significantly affected by the m.3243A>G mutation (14/17 with and 10/22 without the mutation; *p* = 0.093, Fisher's exact test; Fig. 3A). However, presence of this mutation significantly increased both the worst pain intensity (*p* = 0.043, Wilcoxon test; Fig. 3D; Fig. A.3D–F) and the likelihood of neuropathic pain compared to all other mutations (SFNSL: *p* = 0.027, *t*-test; painDETECT: *p* = 0.0070, Wilcoxon test; Fig. 3B,C). Apart from bodily pain and general health, most physical and mental function measures were not significantly affected by presence of the m.3243A>G mutation either (Fig. 3E), and neither was age of respondents (49.1 ± 16.0 years with and 45.6 ± 16.4 years without the m.3243A>G mutation; *p* = 0.58, Wilcoxon test). This suggests that the effect on pain of the m.3242A>G mutation could be independent of overall disease severity. Our data thus indicate that distribution, intensity and type of pain are genetically determined in patients with mitochondrial disorders.

## 4 Discussion

Our findings show that chronic pain is prevalent among patients with mitochondrial disease and its presence is significantly higher than the general population. Neuropathy is a likely contributing factor, as indicated by the distribution of the affected dermatomes and the overall high scores on the PainDETECT and SFNSL questionnaires. Neuropathy in the context of mitochondrial disease is thought to be more associated with specific nuclear gene defects, in particular POLG mutations [5,11]. However, in our survey, patients with nuclear gene mutations did not report more pain than those with mtDNA defects; the sample size was not large enough to compare individual gene defects. We did find that the mt.3243A>G point mutation in the mitochondrial genome was associated with significantly more pain, which was predominantly of neuropathic character. Given the emerging roles for mitochondria in inflammatory responses, for example through the release of mtDNA [12], it will be interesting to see whether chronic inflammation could further contribute to these pain symptoms. Unexpectedly, the presence of chronic pain did not clearly impact on the overall quality of life measures, unlike other types of chronic pain, such as low back pain [13]. This could be due to the small sample size and requires further investigation in future studies.

Aside from the relatively small sample size, the main limitations of our survey include its retrospective nature, the inherent difficulties with patient self-assessment, and the absence of a matched control population with chronic pain but without mitochondrial disease. The impact on quality of life, the nature and the genetic origin of this pain all require more attention in future research into mitochondrial disease. Optimal treatment strategies also need further exploration. Mitochondrial toxicity has been reported for several anti-epileptic drugs that are used in the management of neuropathic pain [14]. However, this is not known to be the case in vivo [15] and in absence of specific evidence, we currently treat neuropathic pain symptoms with conventional pain medications, but only after thorough clinical assessment [16]. Guidelines for neuropathic pain management have been developed, for example by NICE in the United Kingdom [8] and non-pharmacological and psychological approaches should be offered where possible [16,17]. The findings of this survey merit further investigation and have implications for the management of adult patients with mitochondrial disease. We would suggest clinicians screen and treat pain-related symptoms as part of the standard management of patients with mitochondrial disease.

## Appendix Supplementary materials

Image, application 1

Image, application 2

## References

[1] Gorman G.S., Schaefer A.M., Ng Y., Gomez N., Blakely E.L., Alston C.L. (2015). Prevalence of nuclear and mitochondrial DNA mutations related to adult mitochondrial disease. Ann Neurol.

[2] Parikh S., Goldstein A., Karaa A., Koenig M.K., Anselm I., Brunel-Guitton C. (2017). Patient care standards for primary mitochondrial disease: a consensus statement from the mitochondrial medicine society. Genet Med.

[3] Nightingale H., Pfeffer G., Bargiela D., Horvath R., Chinnery P.F (2016). Emerging therapies for mitochondrial disorders. Brain.

[4] Filosto M., Cotti Piccinelli S., Lamperti C., Mongini T., Servidei S., Musumeci O. (2019). Muscle pain in mitochondrial diseases: a picture from the Italian network. J Neurol.

[5] Mancuso M., Orsucci D., Angelini C., Bertini E., Carelli V., Pietro Comi G (2016). “Mitochondrial neuropathies”: a survey from the large cohort of the Italian network. Neuromuscul Disord.

[6] Kraya T., Deschauer M., Joshi P.R., Zierz S., Gaul C (2018). Prevalence of headache in patients with mitochondrial disease: a cross-sectional study. Headache J Head Face Pain.

[7] Bridges S. Chronic pain. Health survey for England - 2011. 2012.

[8] National Institute for Health Care and Exellence (2013). Neuropathic pain in adults : pharmacological management in non- specialist settings. NICE Guidel.

[9] Gorman G.S., Chinnery P.F., DiMauro S., Hirano M., Koga Y., McFarland R. (2016). Mitochondrial diseases. Nat Rev Dis Prim.

[10] Pareyson D., Piscosquito G., Moroni I., Salsano E., Zeviani M (2013). Peripheral neuropathy in mitochondrial disorders. Lancet Neurol.

[11] Horga A., Pitceathly R.D.S., Blake J.C., Woodward C.E., Zapater P., Fratter C. (2014). Peripheral neuropathy predicts nuclear gene defect in patients with mitochondrial ophthalmoplegia. Brain.

[12] West A.P., Shadel G.S. (2017). Mitochondrial DNA in innate immune responses and inflammatory pathology. Nat Rev Immunol.

[13] Luo X., George M.L., Kakouras I., Edwards C.L., Pietrobon R., Richardson W. (2003). Reliability, validity, and responsiveness of the short form 12-item survey (SF-12) in patients with back pain. Spine.

[14] Finsterer J. (2017). Toxicity of antiepileptic drugs to mitochondria. Handb Exp Pharmacol.

[15] Orsucci D., Caldarazzo Ienco E., Siciliano G., Mancuso M (2019). Mitochondrial disorders and drugs: what every physician should know. Drugs Context.

[16] Gilron I., Baron R., Jensen T (2015). Neuropathic pain: principles of diagnosis and treatment. Mayo Clin Proc.

[17] Turk D.C., Swanson K.S., Tunks E.R (2008). Psychological approaches in the treatment of chronic pain patients - when pills, scalpels, and needles are not enough. Can J Psychiatry.

