# Supplementary material for: Chronic pain is common in mitochondrial disease
Source: Neuromuscul Disord. 2020 May;30(5):413–9. doi: 10.1016/j.nmd.2020.02.017 (PMC7306151; doi:10.1016/j.nmd.2020.02.017)
Supplement: Supplementary file 2 [file mmc2.pdf]

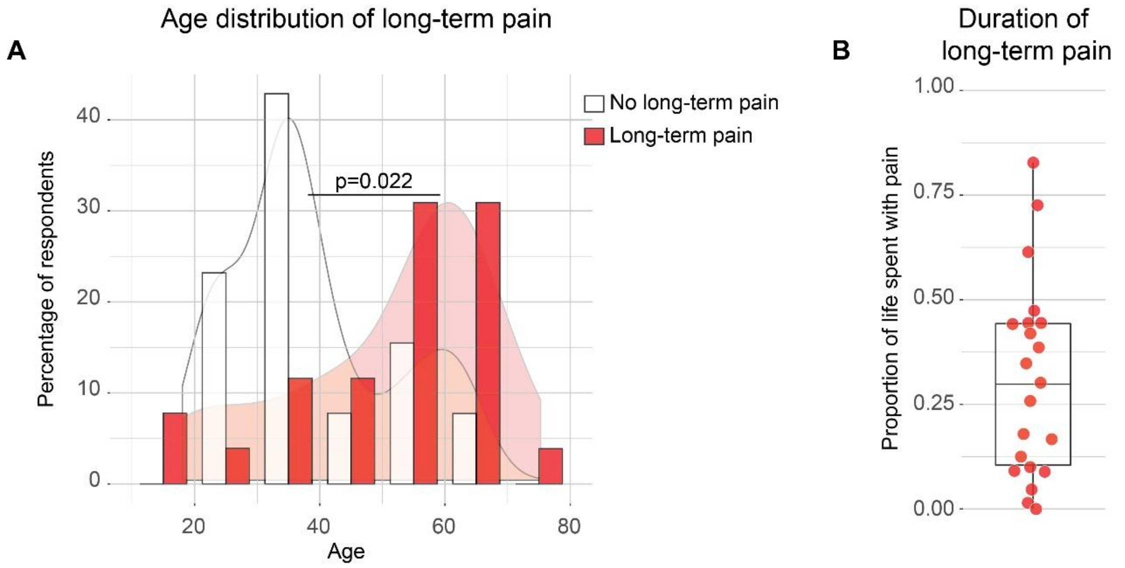

Supplementary figure A.1. Patients with mitochondrial disease experience chronic pain. (A) Age distribution of respondents who reported chronic pain (red) or no chronic pain (white) (p=0.022; Wilcoxon test). (B) The proportion of their life that respondents who report chronic pain spent with this pain ((current age - age of onset)/current age). Box-and-whisker plot depicts median, interquartile range (box) and 1.5IQR below and above the first and third quartiles respectively (whiskers). Datapoints indicate individual responses.

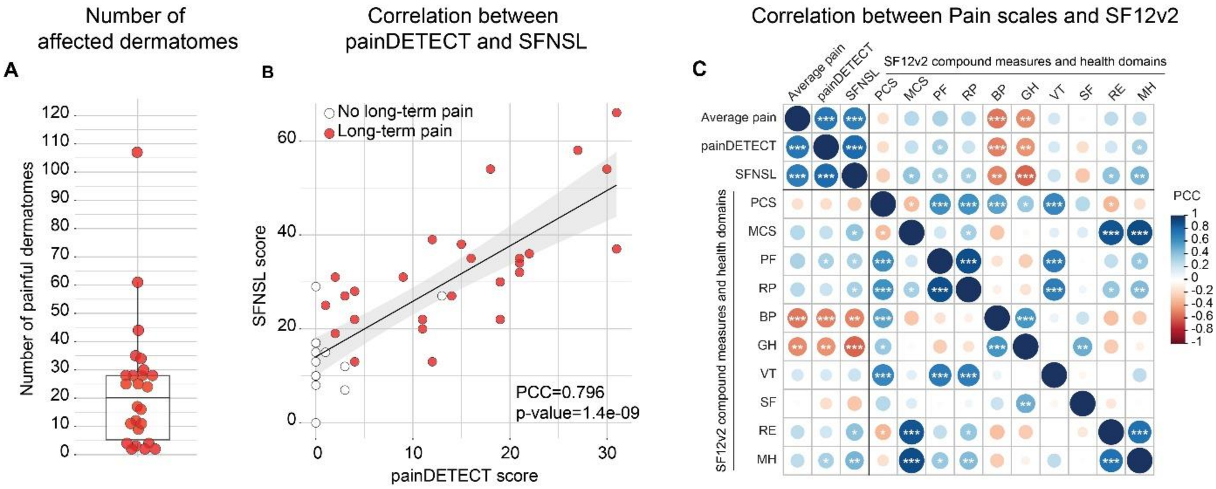

Supplementary figure A.2. Chronic pain is often of neuropathic character. (A) Number of dermatomes in which chronic pain is reported on the body chart by respondents who experience chronic pain. (B) Correlation between painDETECT and SFNSL scores. PCC: Pearson’scorrelation coefficient. Datapoints indicate individual responses. (C) Correlation matrix between average pain intensity, PainDETECT, SFNSL scores and norm-based measures of the SF12v2. The colour scale shows the Pearson Correlation Coefficient (PCC); size of the circle reflects statistical significance, with non-adjusted p-value intervals indicated by the number of asterisks; * ($p<0.05$), ** ($p<0.01$), *** ($p<0.001$). PCS, physical component score; MCS, mental component score; PF, physical functioning; RP, role physical; BP, bodily pain; GH, general health; VT, vitality; SF, social functioning; RE, role emotional; MH, mental health.

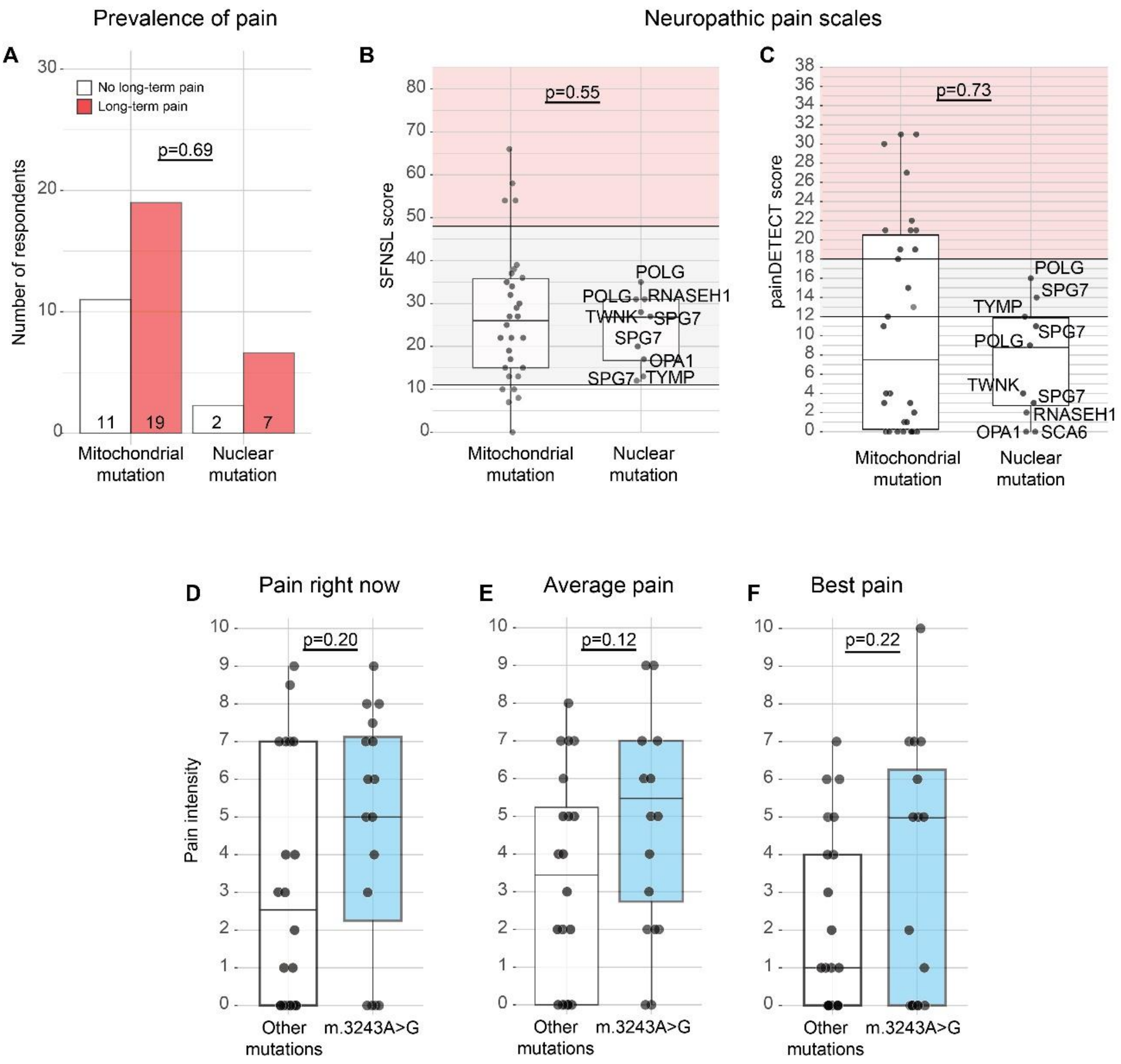

Supplementary figure A.3. Association between pain symptoms and the underlying genetic mutation. (A) Number of respondents with (red) or without (white) chronic pain who have a mitochondrial or a nuclear mutation as the cause of their mitochondrial disease (Fisher's exact test). (B,C) SFNSL (B) and painDETECT (C) scores in respondents with nuclear or mitochondrial mutations (t-test, B; Wilcoxon test, C). Grey and red shaded areas indicate possible and likely neuropathic pain respectively. (D-F) Current (D), average (E) and best (F) pain intensity in patients with (blue) or without (white) the m.3243A>G mitochondrial mutation (Wilcoxon test). Box-and-whisker plots depict median, interquartile range (box) and 1.5IQR below and above the first and third quartiles respectively (whiskers). Datapoints indicate individual responses.

Patient	Mutation	Gender	Age
040*	m.3243A>G	F	52
041	m.3243A>G	F	58
042	m.3243A>G	F	37
043	m.13513G>A	M	60
044	POLG	F	71
045	Unknown	Unknown	Unknown
046	TYMP	F	28
047	Unknown	Unknown	Unknown
048	m.3243A>G	F	49
049	RRM2B	F	33
050	m.7512T>C	F	34
051	RRM2B	F	38
052	m.3243A>G	F	20
053	m.3243A>G	F	24
054	OPA1	F	19
055	Unknown	Unknown	Unknown
056	Unknown	Unknown	Unknown
057	m.11778A>G	M	40
058	m.3243A>G	F	40
059	Unknown	Unknown	Unknown
060	m.3243A>G	F	19
061	m.3460G>A	F	49
062	Single deletion	F	51
063	Unknown	Unknown	Unknown
064	Unknown	Unknown	Unknown
065	Unknown	Unknown	Unknown
066	Unknown	Unknown	Unknown

Supplementary table A.1. Demographics and genetic diagnosis of non-responders. Questionnaires were handed out during the clinic as part of a service evaluation, and 9 of these questionnaires were lost to follow-up (Unknown). Non-responders were on average younger (38.2±15 years) than responders (47.1±16.1 years), but not significantly (p=0.072; Wilcoxon test). *Patient 040 did return the survey, but question 1 (Have you had long-term pain in the past 6 months?) was not completed.

Supplementary table A.2. Overview of all answers to the survey. The table is provided as a separate .xlsx file and includes all questions that were part of the survey. Answers are coded according to the scoring manuals of each respective questionnaire.
